# Supplementary material for: Causal Relationship between Aging and Anorexia Nervosa: A White-Matter-Microstructure-Mediated Mendelian Randomization Analysis
Source: Biomedicines. 2024 Aug 16;12(8):1874. doi: 10.3390/biomedicines12081874 (PMC11351342; doi:10.3390/biomedicines12081874)
Supplement: Supplementary file 1 [file biomedicines-12-01874-s001.zip › Supplementary Figure/Figure S8/Leaveoneout plot of MR analysis for TL to ubm-b-2084.pdf]

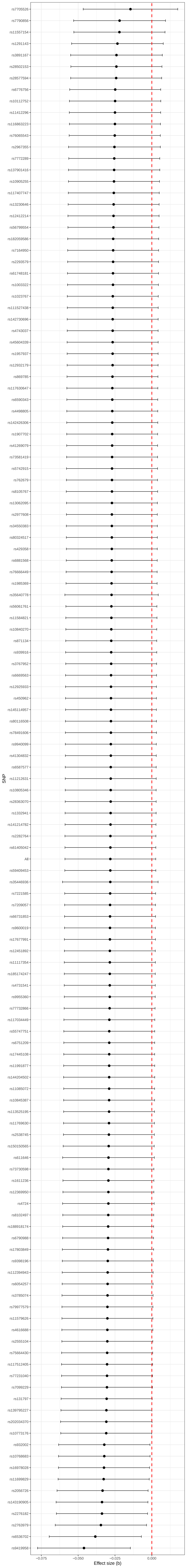
rs7705526
rs1790856
rs11557154
rs1291143
rs3891167
rs28502153
rs28577594
rs6776756
rs10112752
rs11412296
rs116863223
rs76065543
rs2967355
rs7772289
rs137901416
rs10905255
rs117407747
rs13230646
rs12412214
rs56799554
rs182059586
rs7164950
rs2293579
rs61748181
rs1003322
rs1023767
rs111527438
rs142730696
rs4743037
rs45604339
rs1957937
rs12932179
rs869785
rs117630647
rs6590343
rs4498805
rs142426306
rs1907702
rs41269079
rs73581419
rs5742915
rs762679
rs8105767
rs13062095
rs2977608
rs34550383
rs80324517
rs429358
rs6881568
rs76666449
rs1985369
rs35640778
rs56061761
rs11584821
rs10840270
rs871134
rs939916
rs3767952
rs6669563
rs12925933
rs450962
rs145114957
rs80116508
rs78491606
rs9940099
rs41304832
rs6587577
rs11212631
rs10805346
rs28363070
rs1332941
rs141214782
rs2282764
rs61405042
All
rs59409453
rs35446936
rs7221585
rs7209057
rs66731853
rs9600019
rs17677991
rs12451892
rs11117354
rs185174247
rs4731541
rs9955360
rs77732866
rs117034449
rs55747751
rs6751209
rs17445108
rs11991877
rs144204502
rs11085072
rs10845387
rs113525195
rs11769630
rs2538745
rs150150565
rs611646
rs73730598
rs1611236
rs12369950
rs4724
rs8102497
rs188918174
rs6790988
rs17803849
rs9398196
rs112394943
rs6054257
rs3785074
rs79977579
rs11579626
rs4616688
rs2555104
rs75664430
rs117512405
rs77231040
rs7099229
rs131797
rs139795227
rs202034370
rs10773176
rs932002
rs10768683
rs16978028
rs11699829
rs2056726
rs143190905
rs2276182
rs2763979
rs6536702
rs9419958
SNP
-0.075
-0.050
-0.025
0.000
Effect size (b)
